# Effect of Alteration in Precipitation Amount on Soil Microbial Community in a Semi-Arid Grassland

**DOI:** 10.3389/fmicb.2022.842446

**Published:** 2022-03-17

**Authors:** Junyong Li, Girmaye Benti, Dong Wang, Zhongling Yang, Rui Xiao

**Affiliations:** ^1^School of Life Sciences, Henan University, Kaifeng, China; ^2^College of Geography and Environmental Science, Henan University, Kaifeng, China

**Keywords:** bacterial and fungal communities, community composition, changing precipitation, diversity, grasslands

## Abstract

Climate models predict significant changes in precipitation magnitude in semi-arid grasslands, so it is vital to improve our understanding of how changing precipitation affects microbial communities in grassland ecosystems. Using a long-term field manipulation experiment, we evaluated the responses of microbial communities to a decrease (DP) and an increase (IP) in precipitation on a semi-arid grassland in northern China. The results showed that bacterial species richness decreased significantly with DP but remained stable under IP. Relative abundance of oligotrophic, slow-growing bacterial phyla (e.g., Chloroflexi and Acidobacteria) increased with DP but decreased with IP, whereas the relative abundance of copiotrophic, fast-growing bacterial phyla (e.g., Proteobacteria and Bacteroidetes) decreased with DP but increased with IP. In contrast, diversity, species composition, and relative abundance of different fungal phyla change little with DP or IP. These results indicate a greater sensitivity of bacteria to precipitation changes than fungi, and the sensitivity of bacteria to DP was higher than IP. Our findings are important for understanding soil microbial dynamics under future climate change scenarios.

## Introduction

Climate change is affecting precipitation patterns and causing changes in the amount of precipitation (Huntington, 2006). Changes in the amount of precipitation greatly affect soil microbes and the biogeochemical pathways they control, especially in semi-arid grasslands where water availability is increasingly important for regulating microbial community dynamics (Nielsen and Ball, 2015). However, limited knowledge is available on how altering soil water and nutrient availability induced by precipitation changes regulating microbial communities (Evans and Wallenstein, 2014).

The effects of change in the amount of precipitation on microbial communities have been well-reported (Clark et al., 2009; Landesman and Dighton, 2010; Nielsen and Ball, 2015; Gao et al., 2016; Zhou et al., 2018). However, experimental results have been highly variable. Some studies have found that drought shifts the bacterial community toward Gram-positive bacteria (with strong, thick, interlinked peptidoglycan cell walls) because they are inherently resistant to dry conditions (Uhlirová et al., 2005; Zhou et al., 2018). In contrast, no changes in species composition, either in bacterial or fungi communities after decreases in precipitation, have been demonstrated in tallgrass prairie (Williams, 2007). Fungi tend to be drought-tolerant due to their ability to accumulate osmoregulatory solutes to protect their metabolism and filamentous structure; these properties allow fungi to exploit substrates in very dry soils (Schimel et al., 2007; Nielsen and Ball, 2015). Therefore, it is necessary to examine how changes in precipitation amount affect bacterial groups, fungal groups, and different phyla of bacteria and fungi that display contrasting water-related life strategies.

Abiotic factors such as soil temperature (Feng and Simpson, 2009), soil moisture (Brockett et al., 2012), and soil pH (Fierer and Jackson, 2006; Lauber et al., 2009) are major drivers of microbial communities. Changes in precipitation could directly influence soil microbes by altering soil water availability or indirectly by regulating nutrient availability and soil temperature (Brockett et al., 2012; Koyama et al., 2017). Plant communities can also affect microbial communities by regulating microclimatic variability, resource availability, and habitat complexity (Waldrop et al., 2006; Koyama et al., 2017). Maestre et al. (2015) showed that drought reduced diversity and abundance of soil bacteria and fungi by decreasing plant cover and consequent organic carbon inputs into the soil. Changes in plant diversity can lead to changes in plant products and diversity of litter organic components, thus affecting the composition and function of soil microbial communities (Spehn et al., 2000). However, Meier and Bowman (2008) have found that soil microbial diversity is positively correlated with the diversity of chemical components produced by plant litters while there is no direct correlation with the diversity of plant species.

Semi-arid grasslands are very sensitive to changes in soil water availability, and changes in precipitation have been reported to affected plant species diversity and community composition (Zhang et al., 2020a). However, how changes in precipitation shift microbial communities and the drivers of microbial communities are still unclear due to the complexity of soil ecosystem and the limitation of soil microbial research techniques. Here, we performed a manipulation experiment in a semi-arid steppe in northern China to answer the following two questions: (1) how do increases (IP) and decreases (DP) in precipitation change the bacterial and fungal groups? (2) which factors drive the alterations in microbial communities under changing precipitation?

## Materials and Methods

### Study Site

The study site is located in a typical temperate steppe at the Duolun Restoration Ecology Station (42°02′N, 116°16′E, 1324 m a.s.l.) in Inner Mongolia, China. Mean annual precipitation from 1961 to 2014 in the area is ∼383 mm with more than 90% occurring in the growing season (from May to October). Mean annual temperature is 2.1°C, with a monthly mean temperature of −17.5°C in January and 18.9°C in July (Yang et al., 2020).

### Experimental Design

We used a randomized block design with three treatments: ambient precipitation (C), a 60% decrease in growing season precipitation (from April to October, DP), and a 60% increase in growing season precipitation (from April to October, IP). Each treatment had five replicates giving 15 plots in total. Each plot had an area of 16 m^2^ (4 m × 4 m), and was randomly distributed in each block. Buffer zones between plots were 1.5 m wide (Zhang et al., 2020a). Precipitation was blocked using shelters in the DP plots when IP plots received additional 60% precipitation with a handheld irrigation system. The experiment was established on April 15, 2015.

### Vegetation Monitoring

The above-ground vegetation was sampled in 1 m × 1 m quadrat in each plot at the end of September when plant biomass reached its peak level (Sagar et al., 2017). Species richness and abundance were estimated, and individual plants in each quadrat were clipped to the soil surface. Biomass was then sorted by species, and number of the species was also recorded for each quadrat. All shoot samples were dried at 80°C for 48 h, and weighed (Liang et al., 2013).

### Soil Moisture and Soil Temperature Measurement

The soil temperature (ST, 0–10 cm) was measured using a temperature probe, an accessory to the Li-8100 (Li-Cor, Inc., Lincoln, NE, United States) on September 10, 2018 (Sagar et al., 2017). Volumetric soil water content (SM, 0–10 cm) was measured with a portable device (Diviner 2000, Sentek Pty Ltd., Balmain, NSW, Australia).

### Soil Sampling and Analysis

The soil samples were collected on September 10, 2018, at depth 0–20 cm. Six soil cores (approximately 5 cm diameter and 20 cm in depth) were taken from each plot and mixed to form one composite sample. Each sample was placed in a sterile plastic bag, sealed, and placed on ice when transported to the laboratory. Soil samples were sieved through a 2.0 mm mesh and stored at 4°C for analysis of soil characteristics or subsamples at -80°C for DNA extraction. The concentrations of NH_4_^+^ and NO_3_^–^ were detected using a flow injection analyser (Tecator Inc., Hoganas, Sweden) (Miao et al., 2019). Soil total C (TC) and N (TN) contents were determined on a Vario MICRO cube elemental analyzer (Elementar, Germany). The soil total P (phosphorus, TP) was analyzed using the Olsen methods (Long et al., 2020).

### DNA Extraction and Sequencing

DNA was extracted from 0.3 g fresh soil of each sample using MoBio Power soil TMDNA isolation kits (San Diego, CA, United States) according to the manufacturer’s instructions. Primer sets ITS1F (CTTGGTCATTTAGAGGAAGTAA)/ITS2R (GCTGCGTTCTTCATCGATGC) and 338F (ACTCCTACG GGAGGCAGCAG)/806R (GGACTACHVGGGTWTCTAAT) were selected to amplify the genes of fungi ITS and bacterial 16SrRNA, respectively. The PCR products for fungal and bacterial genes were gel purified and further quantified using PicoGreen Kits (Invitrogen, Shanghai, China). Sequencing was performed using the Illumina MiSeq platform at Majorbio Company (Shanghai, China). The raw high-throughput sequencing data were first processed using the Quantitative Insights Into Microbial Ecology (QIIME) toolkit. The potentially similar sequences were clustered into operational taxonomic units (OTUs) at a similarity level of 97%. Taxonomic assignment was performed by blasting the representative sequences against the MaarjAM (fungi) and SILVA (bacteria) database. Finally, this resulted in a total of 991,215 bacterial sequences and 1,132,095 fungal sequences for all samples, respectively. The rarefaction curves for the observed OTUs for both bacteria and fungi reached saturation for each treatment (Supplementary Figure 1), suggesting that the analyzed reads were sufficient to detect most of sequence types. All sequences have been submitted to the GenBank Sequence Read Archive with accession number MN834158–MN836334, KDPW01000000.

### Data Analysis

The sequence data was analyzed on the Majorbio I-Sanger Cloud Platform.^1^ In order to examine how DP and IP affect community composition of plant, bacteria, and fungi, dissimilarity in compositions between treatments were calculated with the non-metric multidimensional scaling (NMDS) analysis using the Bray-Curtis distance matricesbased on plant species, bacterial and fungal OTUs. NMDS values were computed using the “metaMDS” function in the R package vegan v. 2.2–0 (Oksanen et al., 2014). A mixed-effects model analysis was used to test effects of DP and IP on community composition, species richness, and relative abundances of bacterial and fungal, where DP and IP were viewed as fixed between-subjects effect, and block was viewed as random variable. LSD *post hoc* tests were used to test for significant differences in these variables among C, DP, and IP treatments. The analyses were conducted using SPSS 16.0 (SPSS, Inc., Chicago, IL, United States). In order to explore the drivers of different phyla, a heat map analysis was used to examine potential correlations between ST, SM, plant species richness, aboveground net primary productivity (ANPP), NH_4_^+^ and NO_3_^–^ concentration, total nitrogen (TN), total carbon (TC), soil pH, ratio of carbon to nitrogen (C/N), soil TP, and plant community composition with the observed bacterial group (abundance) patterns. The heat map was constructed using the pheatma package of R software 4.0.0.

## Results

### Soil and Plant Attributes

Mean soil moisture was 6.4% in the control plots. DP significantly reduced soil moisture by an average of 1.3%, whereas IP significantly increased soil moisture by an average of 1.3% (Table 1). Both TN and TC were significantly reduced by IP, but remained stable under the DP treatment. DP significantly increased NO_3_^–^ concentration, whereas IP had little effect on it. Neither DP nor IP affected the NH_4_^+^ concentration compared with control (Table 1).

**TABLE 1:** Results (*F*-values) of one-way ANOVAs on effects of decreasing precipitation (DP) and increasing precipitation (IP) on soil temperature (ST, °C), soil moisture (SM, %), NH_4_^+^ (mg kg^–1^), NO_3_^–^ (mg kg^–1^), total nitrogen (TN, mg kg^–1^), total carbon (TC, mg kg^–1^), the ratio of carbon to nitrogen (C/N), phosphorus content (P, mg°kg^–1^), soil pH, plant species richness (m^–2^), aboveground net primary productivity (ANPP, g m^–2^ year^–1^), and plant community composition (The scores of X axis in NMDS).

	ST (°C)	SM (%)	NH_4_^+^ (mg kg^–1^)	NO_3_^–^ (mg kg^–1^)	TN (mg g^–1^)	TC (mg g^–1^)
C	15.5 ± 0.4 a	6.4 ± 0.4b	5.0 ± 0.4ab	5.0 ± 0.2b	1.6 ± 0.0b	14.7 ± 0.5b
DP	16.2 ± 0.6 a	5.2 ± 0.2a	4.5 ± 0.2a	13.4 ± 1.3a	1.5 ± 0.0ab	13.6 ± 0.3ab
IP	15.7 ± 0.4 a	7.7 ± 0.5c	5.5 ± 0.2b	5.4 ± 0.6b	1.5 ± 0.0a	13.3 ± 0.4a

	**C/N**	***P* (mg kg^–1^)**	**pH**	**Plant species richness**	**ANPP (g m^–2^ year^–1^)**	**Plant community composition**

C	9.2 ± 0.1 a	115.5 ± 21.9 a	7.0 ± 0.1 a	18.4 ± 0.9b	77.3 ± 3.3 a	0.08 ± 0.02 a
DP	9.1 ± 0.1 a	104.6 ± 16.5 a	7.2 ± 0.1 a	11.6 ± 2.5a	60.6 ± 3.5 b	−0.25 ± 0.05 b
IP	9.1 ± 0.1 a	151.6 ± 10.1 a	7.2 ± 0.1 a	19.2 ± 1.4b	83.9 ± 11.1 a	0.17 ± 0.03 a

*Means and standard error are shown. Different letters indicate significant difference based on p < 0.05.*

There was 18.4 plant species m^–2^ in the control plots. DP significantly reduced plant species richness by an average of 6.8 species m^–2^, whereas IP had no significant effect. ANPP was 71.6 g m^–2^ year^–1^ in the control plots. DP decreased ANPP by 16.7 g m^–2^ year^–1^, whereas IP had no effect on ANPP. The scores of X axis in NMDS in 2018 data showed that plant species composition in the DP plots diverged from the control plots, whereas composition did not differ in the IP communities from the control plots (Table 1 and Supplementary Figure 2).

### Bacterial and Fungal Diversity

**FIGURE 1:**
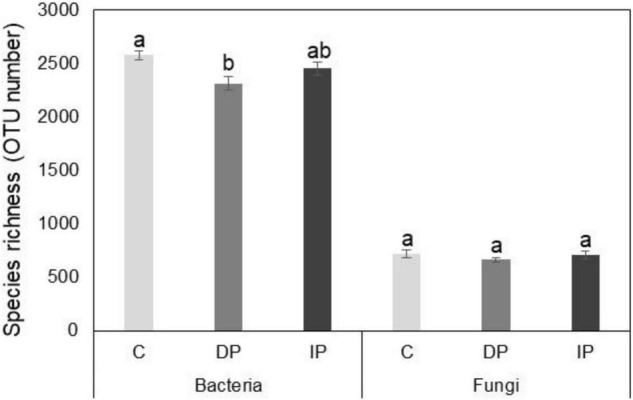
Effect of increasing precipitation (IP) and decreasing precipitation (DP) on species richness of soil bacteria and fungi. C represents control. Different letters indicate significant differences based on *p* < 0.05.

High throughput sequencing yielded a total of 1,177,241 bacterial and 1,315,445 fungal DNA sequences, with 3,444 and 2,142 operational taxonomic units, average read lengths of 439 and 267 bp, and average library coverage of 97.7 and 94.9%, respectively. Species richness of the bacterial communities decreased with DP [*F*_(1,9)_ = 11.9, *P* = 0.009], whereas IP had no significant effect compared to ambient precipitation [*F*_(1,9)_ = 2.7, *P* > 0.05]. Neither DP nor IP affected fungal species richness (Figure 1).

### Community Composition of Bacteria and Fungi

**FIGURE 2:**
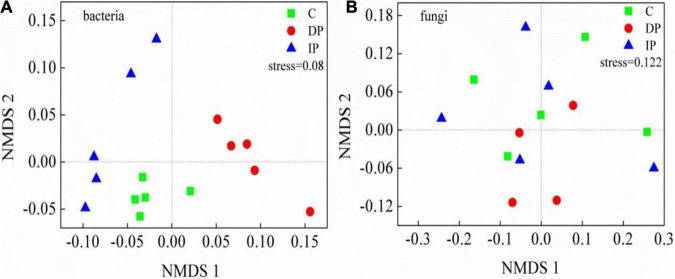
Non-metric multidimensional scaling analysis (NMDS) of soil bacterial and fungal communities under decreasing precipitation (DP) and increasing precipitation (IP). See Figure 1 for abbreviations.

**FIGURE 3:**
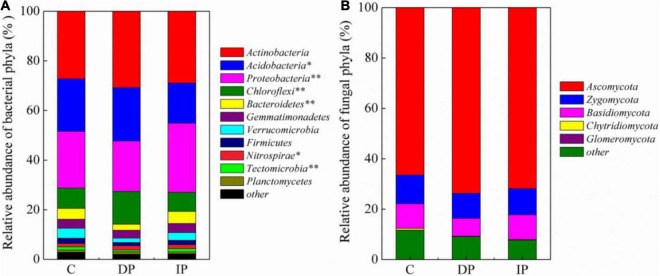
The relative abundances of bacterial **(A)** and fungal **(B)** phyla in the soil samples. See Figure 1 for abbreviations. The value of *p* < 0.05 are marked “*”, the value of *p* < 0.001 are marked “^**^”.

The composition of bacterial communities were significantly changed by both DP and IP (Figure 2), and the top 1% of OTUs loadings on the axis1 in MNDS were shown in Supplementary Table 1. In contrast, neither DP nor IP affected the composition of the fungal community, the top 2% of OTUs loadings on the axis1 in MNDS were shown in Supplementary Table 2. Based on the number of OTUs, we categorized microbal groups at the phyla level. Among the bacteria, the most abundant phyla were Actinobacteria, with 27.2–30.8% of the sequences, followed by Proteobacteria (20.3–27.8%), Acidobacteria (16.2–21.4%), and Chloroflexi (8.2–13.1%). Actinobacteria was stable regardless of DP or IP. DP increased Chloroflexi and Nitrospirae by 59.39% [*F*_(1,9)_ = 50.45, *P* < 0.001] and 33.41% [*F*_(1,9)_ = 9.21, *P* < 0.05], respectively, whereas IP had no effect on either Chloroflexi [*F*_(1,9)_ = 0.7, *P* > 0.05] or Nitrospirae [*F*_(1,9)_ = 0.8, *P* > 0.05]. Acidobacteria decreased 23.40% [*F*_(1,9)_ = 9.84, *P* = 0.01] by IP but was not affected by DP [*F*_(1,9)_ = 0.14, *P* > 0.05]. Bacteroidetes decreased 43.06% [*F*_(1,9)_ = 30.65, *P* = 0.001] by DP but remained stable under IP [*F*_(1,9)_ = 1.50, *P* > 0.05]. In contrast, Proteobacteria increased 21.96% [*F*_(1,9)_ = 14.17, *P* = 0.006] by IP but had little response to DP [*F*_(1,9)_ = 1.89, *P* > 0.05]. Soil fungal communities were dominated by Ascomycota (66.5–73.7%), Zygomycota (9.9–11.3%), and Basidiomycota (7.1–9.8%). However, the relative abundance of the fungal phyla remains stable under either DP or IP (Figure 3).

### Factors Influencing Relative Abundance of Bacterial Phyla

**FIGURE 4:**
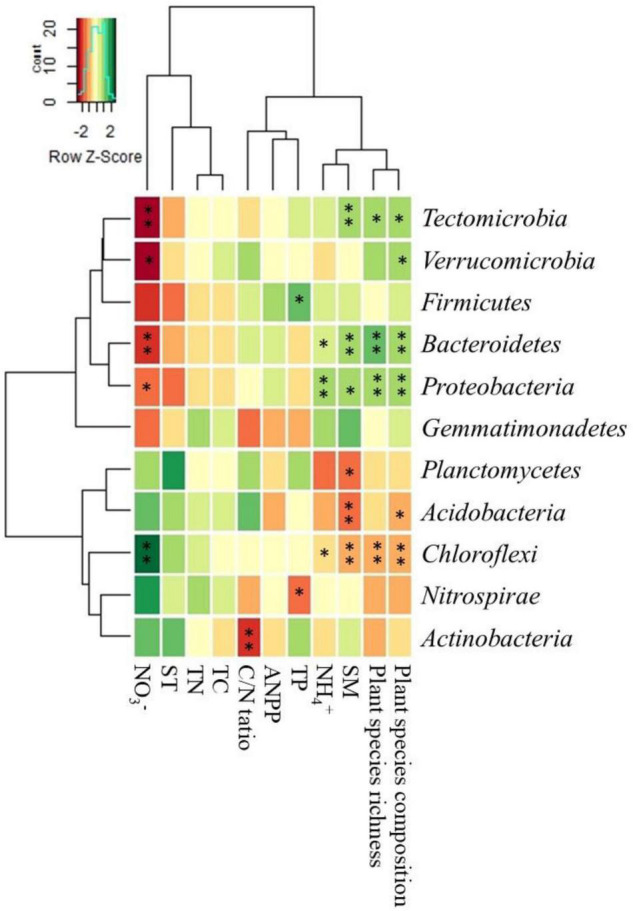
Correlation heat map of soil and vegetation properties with relative abundance of the top eleven bacterial phyla. The right side of the legend is the color range of *R*-values. See Table 1 for abbreviations. The values of *p* < 0.05 are marked with “*.” The values of *p* < 0.001 are marked with “^**^”.

The correlation heatmap showed that the relative abundances of drought-tolerant bacterial phyla (e.g., Acidobacteria and Chloroflexi) showed a significant negative correlation with SM, plant species richness, and ANPP but positively correlated with plant species composition. The relative abundances of drought-sensitive bacterial phyla (e.g., Proteobacteria and Bacteroidetes) were positively correlated with SM, plant species richness, and ANPP but negative correlation with plant species composition (Figure 4).

## Discussion

### Effects of Changing Precipitation on Microbial Communities

Bacteria are more sensitive to changes in precipitation than fungi, particularly to decreases in precipitation (Maestre et al., 2015). Our results show that changes in precipitation influenced bacterial communities rather than fungal communities. Phyla within soil bacterial groups respond differently to changing precipitation due to different evolutionary adaptations and physiological acclimation mechanisms (Cregger et al., 2012). Our study demonstrates that bacteria within the phyla Acidobacteria, Chloroflexi, and Nitrospirae were more abundant under precipitation decreases. These groups are characterized by slow growth rates and mini-colony formation, which are typical characteristics of oligotrophic organisms. These bacterial taxa can maintain their abundance better under dry environments due to a greater tolerance to water stress (Evans and Wallenstein, 2014; Ochoa-Hueso et al., 2018). In contrast, Proteobacteria and Bacteroidetes, which are water-sensitive bacteria, increase under precipitation increases and decrease under precipitation decreases. These groups are generally considered to be copiotrophic, fast-growing bacteria and tend to respond to the altered availability of labile carbon induced by changes in precipitation (Fierer et al., 2009).

Fungi are characterized by a resistant response pattern, with generally stable abundance, species richness, and community composition under both precipitation increases and precipitation decreases. A possible explanation is that fungi have a relatively broad water optimum (compared to bacteria) without significant inhibition of growth (de Vries et al., 2018). Fungi are more drought tolerant than bacteria (except actinomycetes) because their hyphae can transfer water and nutrients from water-filled micropores (de Boer et al., 2005). However, some fungi grow well in wetter conditions. Abundant precipitation generally stimulates plant growth (Zhang et al., 2020b), leading to a large amount of organic matter supplied to the soil. Some fungi are copiotrophs and play an important role in decomposing organic matter, such as cellulose and chitin (Fierer et al., 2009).

### Factors Affecting Microbial Communities

Soil water availability plays important role in structuring bacterial communities (Zhou et al., 2018). Limited soil water availability induced by drought decreases bacterial diversity by decreasing solute mobility and constraining the substrate supply to the decomposers (Ilstedt et al., 2000; Manzoni et al., 2014). In this way, decreases in precipitation directly inhibit Gram-negative bacteria (e.g., Proteobacteria) that are highly sensitive to soil moisture (Fierer et al., 2007; Bardgett et al., 2008; Manzoni et al., 2012). In contrast, Acidobacteria, which belongs to Gram-positive bacteria, are much more water-tolerant (Schimel et al., 2007) because of their stronger cell wall and a potentially more advanced osmoregulatory strategy than Gram-negative bacteria (Harris, 1981). Moreover, microbes provide plant nutrients only when their nutrient needs are met in the process of decomposition of organic matter, thus soil available nitrogen regulates the composition of microbial communities (Hodge et al., 2000). However, increased nitrate-nitrogen under drought stress induced by reduced plant and microbial uptake (Zhou et al., 2018) and/or reduction in denitrification and leaching losses in exposed dry soil (Gómez et al., 2012) suggests that soil available nitrogen has a weak effect on bacterial diversity in the current study. The changes in plant species identity and composition related to drought stress tolerance alter microbial communities by favoring plant associations with mycorrhizal fungi and mutualistic soil bacteria (Mariotte et al., 2017; Rubin et al., 2017). For example, mismatches between plant-microbe partners induced by the change in plant communities under drought create fitness differences among microbial species that reorder their relative abundances in the community by altering rates of population growth (Rudgers et al., 2020).

## Conclusion

Our study shows that fungi were insensitive to either increases or decreases in precipitation. In contrast, changes in precipitation shifted the soil bacterial communities. The relative abundance of oligotrophic, slow-growing bacterial phyla increased in response to DP but decreased under IP. In contrast, the relative abundance of copiotrophic, fast-growing bacterial phyla decreased in response to DP but increased under IP. Effects of water availability, nitrate-nitrogen content, and plant-mediated effects are critical for driving the changes in soil microbial communities under changing precipitation. Our study suggests that changes in the amount of precipitation in the semi-arid grassland play an important role in determining soil bacterial communities under future climate change scenarios.

## Data Availability Statement

The original contributions presented in the study are included in the article/Supplementary Material. Further inquiries can be directed to the corresponding author.

## Author Contributions

RX and ZY proposed the scientific hypotheses and supervised the project. JL, GB, and DW collected data. RX and JL performed data analyses and wrote the draft of the manuscript. All authors contributed to manuscript revision, read, and approved the submitted version.

## Conflict of Interest

The authors declare that the research was conducted in the absence of any commercial or financial relationships that could be construed as a potential conflict of interest.

## Publisher’s Note

All claims expressed in this article are solely those of the authors and do not necessarily represent those of their affiliated organizations, or those of the publisher, the editors and the reviewers. Any product that may be evaluated in this article, or claim that may be made by its manufacturer, is not guaranteed or endorsed by the publisher.

## Footnotes

1
http://www.i-sanger.com

## Abbreviations

C: ambient precipitation
DP: a 60% decrease in growing season precipitation
IP: a 60% increase in growing season precipitation
OTUs: operational taxonomic units
NMDS: non-metric multidimensional scaling
ST: soil temperature
SM: soil moisture
TN: total nitrogen
TC: total carbon
C/N: the ratio of carbon to nitrogen
ANPP: above ground net primary productivity.
