# Supplementary material for: Effect of Alteration in Precipitation Amount on Soil Microbial Community in a Semi-Arid Grassland
Source: Front Microbiol. 2022 Mar 17;13:842446. doi: 10.3389/fmicb.2022.842446 (PMC8969558; doi:10.3389/fmicb.2022.842446)
Supplement: Supplementary file 1 [file Table_1.DOCX]

Table S1. Loadings of bacterial OTUs on each axis in the Nonmetric multidimensional scaling (NMDS). The OTUs were showed in the top 1% of the species scores for axis1.

|  | Phylum | Genus | Axis1 | Axis2 |
| --- | --- | --- | --- | --- |
| OTU2457 | Gemmatimonadetes | norank_f__Gemmatimonadaceae | 0.0285 | -0.0242 |
| OTU2356 | Actinobacteria | norank_o__Gaiellales | 0.0257 | 0.0348 |
| OTU1111 | Acidobacteria | norank_c__Acidobacteria | 0.0257 | -0.0312 |
| OTU3428 | Chloroflexi | norank_c__KD4-96 | 0.0252 | 0.0311 |
| OTU2078 | Nitrospirae | Nitrospira | 0.0247 | -0.0146 |
| OTU1588 | Actinobacteria | norank_c__Actinobacteria | 0.0232 | 0.0308 |
| OTU2298 | Proteobacteria | Sorangium | 0.0221 | -0.0052 |
| OTU29 | Gemmatimonadetes | norank_c__Gemmatimonadetes | 0.0217 | -0.0104 |
| OTU111 | Planctomycetes | unclassified_f__Planctomycetaceae | 0.021 | 0.0391 |
| OTU792 | Actinobacteria | norank_c__Actinobacteria | 0.0202 | 0.0173 |
| OTU1577 | Armatimonadetes | norank_p__Armatimonadetes | 0.0187 | 0.0119 |
| OTU2037 | Proteobacteria | norank_f__Caulobacteraceae | 0.0179 | -0.0068 |
| OTU779 | Chloroflexi | norank_f__Anaerolineaceae | 0.0176 | 0.0155 |
| OTU609 | Acidobacteria | Bryobacter | 0.0171 | 0.0019 |
| OTU1543 | Chloroflexi | norank_o__C0119 | 0.0166 | 0.0041 |
| OTU1184 | Actinobacteria | norank_o__Acidimicrobiales | 0.0165 | 0.0144 |
| OTU2656 | Chlorobi | norank_f__OPB56 | 0.015 | -0.0405 |
| OTU453 | Actinobacteria | norank_o__Acidimicrobiales | 0.0144 | -0.0263 |
| OTU334 | Bacteroidetes | unclassified_f__Cytophagaceae | 0.0143 | -0.037 |
| OTU2432 | Proteobacteria | Pedomicrobium | 0.0143 | -0.0051 |
| OTU2041 | Proteobacteria | norank_f__0319-6G20 | 0.0131 | -0.0019 |
| OTU708 | Chloroflexi | norank_f__AKIW781 | 0.0122 | 0.0068 |
| OTU3022 | Actinobacteria | Iamia | 0.0111 | 0.0067 |
| OTU3287 | Chloroflexi | unclassified_p__Chloroflexi | 0.0109 | 0.0039 |
| OTU1503 | Proteobacteria | norank_f__P3OB-42 | 0.0109 | -0.0031 |
| OTU2462 | Acidobacteria | norank_c__Acidobacteria | 0.0108 | 0.0163 |
| OTU2490 | Actinobacteria | norank_c__Actinobacteria | 0.0103 | -0.006 |
| OTU3034 | Sphingobacteriia | unclassified_f__Chitinophagaceae | -0.0105 | 0.0067 |
| OTU613 | Cyanobacteria | unclassified_g__Nostoc | -0.0106 | 0.0078 |
| OTU455 | Acidobacteria | unclassified_g__RB41 | -0.0112 | 0.0019 |
| OTU2805 | unclassified_k__norank | unclassified_k__norank | -0.0112 | 0.0397 |
| OTU469 | Phycisphaerae | uncultured_bacterium_g__CL500-3 | -0.0114 | 0.0025 |
| OTU574 | Acidobacteria | unclassified_g__norank_c__Acidobacteria | -0.0135 | -0.0192 |
| OTU2581 | Cytophagia | uncultured_Bacteroidetes_bacterium_g__Rhodocytophaga | -0.014 | 0.0116 |
| OTU2015 | Acidobacteria | unclassified_g__RB41 | -0.0141 | 0.0158 |
| OTU2376 | Spartobacteria | uncultured_bacterium_g__Chthoniobacter | -0.0149 | -0.0031 |
| OTU3048 | Gemmatimonadetes | unclassified_f__Gemmatimonadaceae | -0.0154 | 0.0088 |
| OTU241 | norank_p__FBP | uncultured_soil_bacterium_g__norank_p__FBP | -0.0177 | 0.0128 |
| OTU2813 | norank_p__Saccharibacteria | uncultured_soil_bacterium_g__norank_p__Saccharibacteria | -0.018 | 0.011 |
| OTU1000 | Anaerolineae | uncultured_Gemmatimonadetes_bacterium_g__Anaerolineaceae | -0.0186 | 0.01 |
| OTU3380 | Fimbriimonadia | uncultured_bacterium_g__norank_f__Fimbriimonadaceae | -0.0187 | -0.0102 |
| OTU3256 | Verrucomicrobiae | unclassified_f__Verrucomicrobiaceae | -0.0187 | -0.0102 |
| OTU2671 | Planctomycetacia | unclassified_f__Planctomycetaceae | -0.0187 | 0.0028 |
| OTU3192 | Cyanobacteria | unclassified_c__Cyanobacteria | -0.0194 | -0.0078 |
| OTU1008 | norank_p__Omnitrophica | unclassified_g__norank_p__Omnitrophica | -0.0199 | 0.0045 |
| OTU3023 | Elusimicrobia | unclassified_g__norank_o__Lineage_IIa | -0.0206 | -0.0057 |
| OTU2163 | Actinobacteria | unclassified_g__Paenarthrobacter | -0.0215 | -0.0023 |
| OTU321 | Betaproteobacteria | uncultured_bacterium_g__norank_f__Nitrosomonadaceae | -0.022 | -0.0168 |
| OTU2741 | Sphingobacteriia | unclassified_g__norank_f__Chitinophagaceae | -0.0228 | -0.0233 |
| OTU2251 | Flavobacteriia | uncultured_bacterium_g__norank_f__NS9_marine_group | -0.0242 | 0.0011 |
| OTU2224 | Sphingobacteriia | uncultured_bacterium_g__Segetibacter | -0.0279 | 0.005 |
| OTU2349 | OPB35_soil_group | unclassified_g__norank_c__OPB35_soil_group | -0.0297 | -0.0052 |

Table S2. Loadings of fungal OTUs on each axis in the Nonmetric multidimensional scaling (NMDS). The OTUs were showed in the top 2% of the species scores for axis1.

|  | Phylum | Genus | Axis1 | Axis2 |
| --- | --- | --- | --- | --- |
| OTU1780 | Ascomycota | unclassified_p__Ascomycota | 0.0352 | 0.0412 |
| OTU823 | Ascomycota | Phaeomycocentrospora | 0.0352 | -0.0106 |
| OTU108 | Ascomycota | unclassified_f__Nectriaceae | 0.0334 | 0.0088 |
| OTU134 | Ascomycota | unclassified_f__norank_o__Pleosporales | 0.033 | -0.0217 |
| OTU1453 | Ascomycota | Alternaria | 0.0316 | 0.0047 |
| OTU1917 | unclassified_k__Fungi | unclassified_k__Fungi | 0.03 | -0.035 |
| OTU624 | Ascomycota | unclassified_f__norank_o__Pleosporales | 0.0288 | 0.0243 |
| OTU1367 | Basidiomycota | unclassified_o__Sebacinales | 0.0267 | -0.0398 |
| OTU1892 | Ascomycota | Volutella | 0.0254 | -0.0292 |
| OTU2022 | Basidiomycota | Clavicorona | 0.0252 | -0.0387 |
| OTU2021 | unclassified_k__Fungi | unclassified_k__Fungi | 0.0252 | -0.0387 |
| OTU1962 | unclassified_k__Fungi | unclassified_k__Fungi | 0.0252 | -0.0387 |
| OTU538 | Ascomycota | unclassified_f__Sporormiaceae | 0.0247 | 0.0058 |
| OTU502 | unclassified_k__Fungi | unclassified_k__Fungi | 0.0226 | -0.0281 |
| OTU116 | Ascomycota | unclassified_p__Ascomycota | 0.0223 | -0.0224 |
| OTU1918 | unclassified_k__Fungi | unclassified_k__Fungi | 0.0201 | 0.0134 |
| OTU1088 | Ascomycota | Schizothecium | -0.021 | 0.0091 |
| OTU1829 | unclassified_k__Fungi | unclassified_k__Fungi | -0.0211 | -0.0068 |
| OTU729 | Glomeromycota | Glomus | -0.0288 | -0.0106 |
| OTU1053 | Ascomycota | unclassified_o__Geoglossales | -0.0329 | 0.0018 |
| OTU1719 | Ascomycota | unclassified_o__Capnodiales | -0.0381 | 0.038 |
| OTU1418 | Ascomycota | unclassified_p__Ascomycota | -0.0411 | 0.0018 |
| OTU766 | unclassified_k__Fungi | unclassified_k__Fungi | -0.049 | -0.0198 |
| OTU1018 | Glomeromycota | unclassified_f__Glomeraceae | -0.0496 | -0.0034 |
| OTU753 | Ascomycota | unclassified_o__Sordariales | -0.05 | -0.0166 |
| OTU30 | Ascomycota | unclassified_o__Coniochaetales | -0.0521 | -0.0068 |
| OTU676 | Ascomycota | unclassified_p__Ascomycota | -0.0531 | 0.0041 |
| OTU1093 | Ascomycota | unclassified_o__Xylariales | -0.0549 | -0.0073 |
| OTU1052 | Ascomycota | unclassified_c__Sordariomycetes | -0.0555 | -0.0106 |
| OTU1008 | Ascomycota | Cladophialophora | -0.0555 | -0.0106 |
| OTU1001 | Basidiomycota | Kurtzmanomyces | -0.0555 | -0.0106 |
| OTU969 | unclassified_k__Fungi | unclassified_k__Fungi | -0.0555 | -0.0106 |
| OTU965 | unclassified_k__Fungi | unclassified_k__Fungi | -0.0555 | -0.0106 |
| OTU511 | Basidiomycota | Entoloma | -0.056 | -0.0098 |
| OTU1946 | Ascomycota | unclassified_f__norank_o__Helotiales | -0.0582 | -0.006 |
| OTU434 | unclassified_k__Fungi | unclassified_k__Fungi | -0.0647 | -0.0039 |


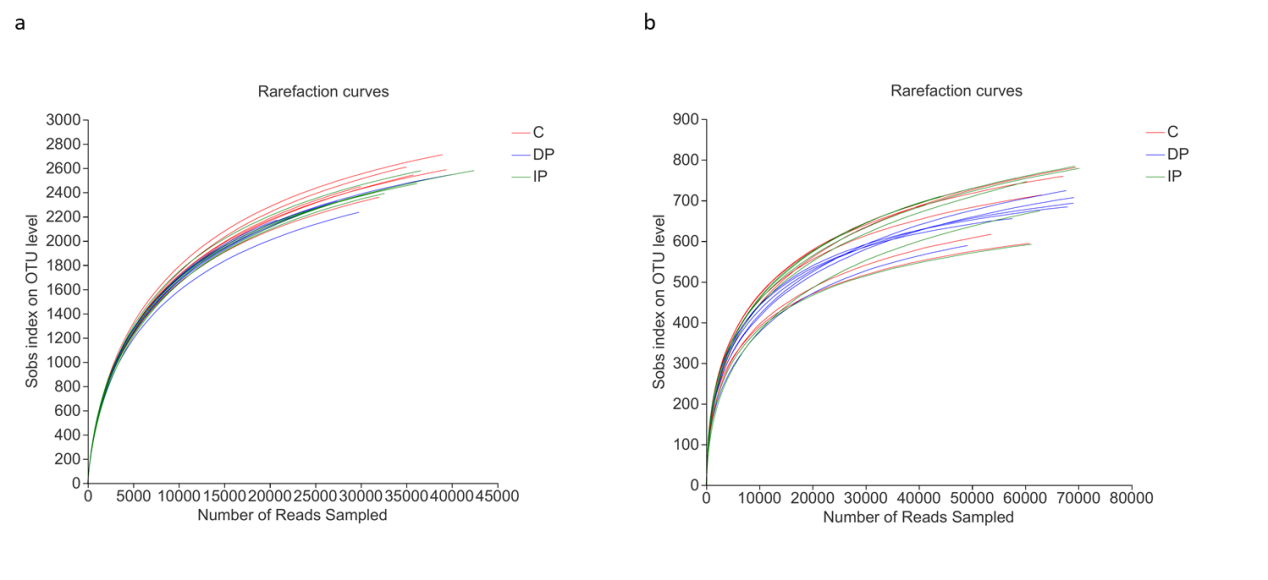


Fig. S1. Rarefaction curves for observed OTUs for bacteria (a) and fungi (b).





Fig. S2. Nonmetric multidimensional scaling (NMDS) patterns of community compositions of plants.
